# A Meta-Analysis of Experimental Studies of Attenuated *Schistosoma mansoni* Vaccines in the Mouse Model

**DOI:** 10.3389/fimmu.2015.00085

**Published:** 2015-02-27

**Authors:** Mizuho Fukushige, Kate M. Mitchell, Claire D. Bourke, Mark E. J. Woolhouse, Francisca Mutapi

**Affiliations:** ^1^Centre for Immunity, Infection and Evolution, College of Medicine and Veterinary Medicine, University of Edinburgh, Edinburgh, UK; ^2^Center for Immunity, Infection and Evolution, Institute of Immunology and Infection Research, School of Biological Sciences, University of Edinburgh, Edinburgh, UK

**Keywords:** schistosomiasis, attenuated cercariae, protective immunity, random effects meta-regression, animal model, systematic review

## Abstract

Schistosomiasis is a water-borne, parasitic disease of major public health importance. There has been considerable effort for several decades toward the development of a vaccine against the disease. Numerous mouse experimental studies using attenuated *Schistosoma mansoni* parasites for vaccination have been published since 1960s. However, to date, there has been no systematic review or meta-analysis of these data. The aim of this study is to identify measurable experimental conditions that affect the level of protection against re-infection with *S. mansoni* in mice vaccinated with radiation attenuated cercariae. Following a systematic review, a total of 755 observations were extracted from 105 articles (published 1963–2007) meeting the searching criteria. Random effects meta-regression models were used to identify the influential predictors. Three predictors were found to have statistically significant effects on the level of protection from vaccination: increasing numbers of immunizing parasites had a positive effect on fraction of protection whereas increasing radiation dose and time to challenge infection had negative effects. Models showed that the irradiated cercariae vaccine has the potential to achieve protection as high as 78% with a single dose vaccination. This declines slowly over time but remains high for at least 8 months after the last immunization. These findings provide insights into the optimal delivery of attenuated parasite vaccination and into the nature and development of protective vaccine induced immunity against schistosomiasis, which may inform the formulation of human vaccines and the predicted duration of protection and thus frequency of booster vaccines.

## Introduction

Schistosomiasis is a water-borne parasitic disease of major public health importance. More than 4.5 million disability adjusted life years (DALYs) are lost each year worldwide due to schistosome infection (1–4). Human schistosomiasis is mainly caused by three species: *Schistosoma mansoni*, *Schistosoma haematobium*, and *Schistosoma japonicum* (5). More than 90% of reported cases are from sub-Saharan Africa where both *S. mansoni* and *S. haematobium* infections are endemic (6). The vast majority of control programs use the antihelminthic drug praziquantel for mass drug administration. This low-cost and efficacious drug has achieved a significant reduction in disease prevalence and infection intensity in many endemic areas (7–10). However, there are multiple reports of re-infection after chemotherapy (11–13). In addition, praziquantel can clear only adult worms and has little or no effect on existing eggs and immature worms (14). This means that there is need for additional complementary interventions, one of which is vaccination.

Slowly developing acquired immunity plays a crucial role in the reduction of infection prevalence and intensity in older age groups in endemic areas (15, 16). This suggests that exposure to schistosome antigens can promote protective immunity in humans; however, to date, there is no licensed schistosome vaccine (17, 18). Currently, the leading vaccine candidate is the 28 kDa *S. haematobium* GST (Sh28GST, Brand name: Bilhvax), which is now in phase 3 clinical trials (19–21). Alongside recombinant antigen vaccine studies, the attenuated live cercariae vaccine has been studied extensively in mouse models (22, 23). Properly prepared attenuated cercariae live long enough to invade the host skin and stimulate protective acquired immunity against subsequent challenge infection but die in the host’s body before they mature into adult worms (24). Attenuated schistosome cercariae vaccination experiments in animals use cercariae, which are weakened by ionizing radiation (X-ray or gamma ray), ultraviolet, heat, or chemical treatment. Host animals are immunized with attenuated parasites either once or several times before challenge infection with non-attenuated pathogenic cercariae. A certain number of days after the challenge infection, immunized animals and control animals are perfused to quantify the level of protection due to immunization by comparing the number of adult worms recovered from both groups.

A large number of mouse experimental studies using attenuated *S. mansoni* cercariae for vaccination have been published since 1960s (25); however, such studies have never been systematically analyzed. The aim of this study was to conduct a meta-analysis to identify measurable experimental conditions (predictors) that affect the level of protection against challenge infection of vaccinated animals. In addition, levels of each predictor associated with maximum levels of protection were estimated.

## Materials and Methods

### Systematic review

An electronic literature search was performed using Science Citation Index Expanded, Conference Proceedings Citation Index and BIOSIS Citation Index, all of which were provided through Web of Knowledge^1^. Alongside these, EMBASE^2^, OVID MEDICINE^3^, and CAB abstracts, were searched simultaneously though OvidSP^4^. Reference lists of all articles identified by the electronic search were searched manually for additional relevant reference. In addition, ProQuest Dissertations and Thesis Full Text^5^ was searched as a source of pre-published and gray literature. The search terms were chosen to be as inclusive as possible and were “cercaria*” AND (“irradiat*” OR “attenuat*”) AND (“vaccin*OR schistosom*”). Also, we searched by “Attenuate*” AND “schistosome*” AND “vaccin*.” This search was completed in July 2013. After duplicated articles were removed a total of 1,013 articles were identified. Titles and abstracts were screened by at least two independent reviewers to exclude those that were not relevant to an attenuated schistosome vaccine animal model. Full texts of potentially relevant articles were reviewed by two independent reviewers for further selection. Non-English articles were included, and several Chinese and German articles were identified and translated into English by a native Chinese speaker and German speaker, respectively, for the analysis.

A study was considered eligible if it met all of the following inclusion criteria: (1) vaccination with attenuated cercariae; (2) use of ionizing radiation for attenuation; (3) use of percutaneous immunization and challenge (i.e., the natural transmission route for schistosome infection); (4) challenge infection using normal (non-attenuated) cercariae; (5) worm burden measured after the challenge infection via perfusion; (6) outcome (fraction of protection) reported or could be calculated. In this study, fraction of protection means the proportion of reduction in worm burden in vaccinated mice compared to that of control mice group. For articles, which reported worm count after challenge infection, the following equation was used to calculate the outcome: fraction of protection = [(average number of worms per mouse retrieved from control group − average number of worms per mouse retrieved from vaccinated group)/average number of worms per mouse retrieved from control group]. In the case of articles, which failed to report worm counts (allowing calculation of this quantity), only those that stated that they used the same equation as above were included.

Studies were excluded if they met any of the following exclusion criteria: (1) immunizing attenuated cercariae developed to adulthood; (2) hosts were transgenic or genetically engineered; (3) hosts had an *in vivo* depletion of immune cells; (4) attenuated cercariae were prepared by any means other than ionizing irradiation; (5) a non-cercarial vaccine was used (e.g., adult worm, schistosomula, subunit); (6) an artificial infection was conducted prior to vaccination; or (7) hosts were treated with anthelmintic drugs.

Articles often reported results from multiple separate experiments such as use of different doses of attenuated parasite. In these cases, results from each experiment were recorded as an observation. A total of 755 observations from 105 articles (articles are listed in Supplementary Material) meeting searching criteria and also using mouse as a host and *S. mansoni* for vaccination and challenge infection. Although the mouse is not a natural host for schistosome infection, it is the most commonly used animal for attenuated schistosome parasite vaccine animal model. A list of potential predictors (given in Table 1) was drawn up and these quantities were extracted from each article. These potential predictors have been suggested their importance by review articles and also their quantities been reported by many experimental studies (26). When an article reported a dose range rather than an exact dose the mid-value was used for the analysis.

**Table 1:** **Possible predictors investigated and their units/codes**.

Variable name	Units/code
Number of immunizing parasites (total and number per dose)	Number of parasites log10 transformed
Number of challenge parasites	Number of parasites log10 transformed
Number of immunizations	Count
Irradiation dose	Krad
Host age	Weeks
Host sex	Male, female, mixed
Time between the last immunization and challenge	Days
Time between challenge and perfusion	Days

### Statistical analysis

#### Random effects meta-regression

Random effects meta-regression was used to identify the influential predictors and effect of dose on protection. Multiple observations (1–56) were recorded from single articles and therefore article was included as a random effect in the models. The models were built using a backwards stepwise procedure with eight potential predictors (listed in Table 1). The effect of the number of immunizing parasites was explored in two ways in the two separate models: as an average number of immunizing parasites per dose or as a total number of immunizing parasites. Correlations between variables were examined visually by scatter plot graphs for all possible predictor combinations (data not shown). Then, all the possible combinations of two-way interactions of potential predictors were examined using a random effects meta-regression model with two-way interactions. The outcome variable (fraction of protection) was transformed as −ln(1 − fraction of protection) to reduce the skewness of residuals (27). Although using confidence intervals and SE is the most common weighting method for meta-regression (28), many studies in our dataset failed to report either confidence intervals or SD and there were no comparable studies, which enabled us to justify imputing them. Two kinds of information were available on the size of the studies: the number of control animals and the number of vaccinated animals. The majority of studies used similar numbers of control and vaccinated animals; however, there were several articles, which used a higher number of vaccinated animals than control animals. To account for the impact of these unbalanced studies, the number of control animals was used as the more conservative weighting option.

#### Missing values and outliers

Several outliers were excluded from the analysis. They were six observations with animals kept longer than 300 days or <7 days after the last immunization and four observations that used more than 10,000 cercariae for immunization. After excluding outliers 745 observations were kept for further selection.

When the numbers of control animals were not reported in an article and only the numbers of vaccinated animals were given, numbers of control animals were then imputed by a linear regression imputation method between numbers of vaccinated and control animals for all studies (29). When the observation was missing for both the number of control animals and vaccinated animals (4 observations from 4 articles), the average number of control animals of the remaining data set was used for imputation, which was 10 control animals. Out of 745 observations, 725 observations from 100 articles reported all predictors and were used for the analysis.

#### Statistical software

Papers identified by systematic review were recorded by Thomson Reuters EndNote and the extracted data were entered on a Microsoft Excel 2010 spread sheet for further analysis. IBM SPSS Statistics Version 19.0 and Minitab. Inc., MINITAB 16 were used for statistical analysis. GraphPad Software GraphPad Prism version 6.03 was used for graphical expression.

## Results

Among eight potential predictors (Table 1), three predictors were found to have statistically significant effects (*P* < 0.05) on the outcome value −ln(1 − fraction of protection) following the backwards stepwise selection: the log10 transformed total number of immunizing parasites (*P* < 0.001), the irradiation dose (*P* < 0.001), and the time between the last immunization and challenge (*P* = 0.04) (Table 2). The reported ranges of each predictor were the total number of immunizing parasites (50–5,000 cercariae), the irradiation dose (3–160 krad), and the time between the last immunization and challenge (7–230 days). All identified predictors were significant (*P* < 0.05) in the model no matter with or without outliers in the model. The number of immunizing parasites was significant in the model regardless of the version of this variable used, i.e., the average number of immunizing parasites per dose or total number of immunizing parasites. In both cases, the models were initially considered with the number of immunizations. When the total number of immunizing parasites was used as a predictor, the number of immunizations was not significant. Therefore, for the final model, the total number of immunizing parasite was used as a predictor with number of immunizations excluded from the model.

**Table 2:** **Results from random effects meta-regression models**.

Predictors	Coefficient	SE	*P*-value
Number of immunizing parasites per dose (log10 transformed)	0.4338	0.0661	<0.001
Irradiation dose	−0.0047	0.0008	0.04
Time between the last immunization and challenge	−0.0015	0.0007	<0.001

*Positive coefficients indicate the predictor’s positive dose effect on fraction of protection whereas negative coefficients indicate predictor’s negative influence on fraction of protection*.

The interaction between log10 transformed total number of immunizing parasites and the time between the last immunization and challenge was statistically significant (*P* = 0.04). However, this interaction was excluded from the final model for the following reasons: (1) the model with the interaction showed biologically implausible fitted values of fraction of protection for some predictors, (2) the model with/without interaction showed similar fitted values for the fraction of protection around the most frequent values of predictors.

**Figure 1:**
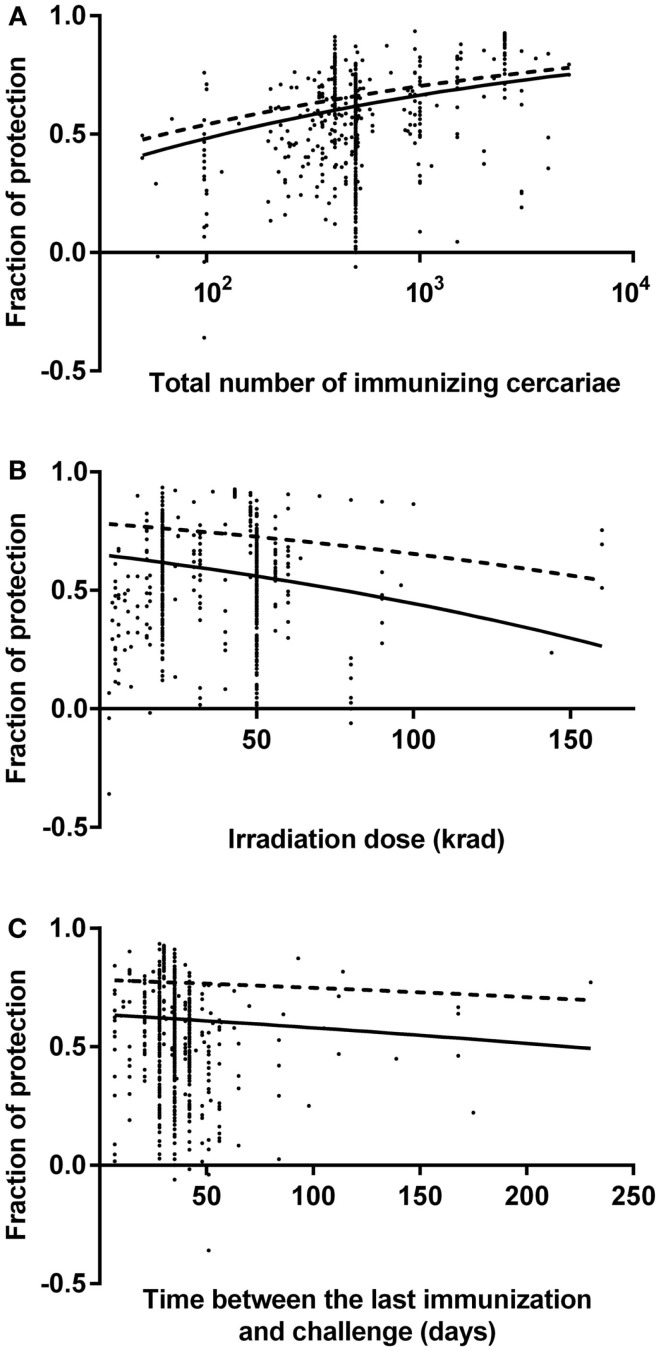
**Fitted graphs for identified predictors from a random effects meta-regression model**. Identified predictors effects on fraction of protection in mouse model: **(A)** the number of immunizing cercariae over the range 50–5,000 cercariae, **(B)** the irradiation dose over the range 3–160 krad, **(C)** the time between the last immunization and challenge over the range 7–230 days. Data points indicate reported fraction of protection for each observation. Negative fractions indicate that vaccination was associated with increase of schistosome worm burden. Lines are fitted graphs generated from random effects meta-regression model (see text). Dashed lines in the graphs show the highest level of protection over range that could be achieved.

Fitted graphs for each predictor are shown in Figure 1 with the outcome variable back-transformed to fraction of protection. Fitted graphs for each predictor were generated by fixing other predictor values at their modes: 500 immunizing parasites, 28 days for the time between the last immunization and challenge, and 20 krad for irradiation dose (solid line in Figure 1). The fitted graph of total number of immunizing parasites and fraction of protection showed the lowest level of predicted protection was 41% with 50 cercariae, which increased up to 75% with 5,000 cercariae (solid line in Figure 1A). Similarly, the minimum level of protection predicted for 160 krad irradiation was 26% protection, which increased to 65% with 3 krad irradiation (solid line in Figure 1B). The estimated level of protection 7 days after the last immunization was 63%, which reduced to 49% by 230 days after the last immunization (solid line in Figure 1C). Fitted graphs showed that the total number of immunizing parasites had a positive impact on the fraction of protection whereas irradiation dose and the time between the last immunization and challenge had negative impacts (Figure 1). Besides this, to estimate the highest protection, fitted graphs for each predictor were generated with other predictor values at their optimal level: 5,000 immunizing parasites, 7 days for the time between the last immunization and challenge, and 3 krad for irradiation dose (dashed line in Figure 1). The models suggested that highest achievable protection was 78% at 7 days after the last immunization, with the mouse immunized with 5,000 cercariae, which were attenuated with 3 krad (dashed line in Figure 1). This 78% protection will decrease over time but would stay as high as 70% by 230 days after the last immunization (dashed line in Figure 1C).

## Discussion

Irradiated *S. mansoni* cercariae vaccines have been tested experimentally against schistosome infection for decades, with important insights obtained from the individual experiments (25). Although the translation of the irradiated parasites vaccine in humans has not been pursued for schistosomiasis, a precedent for this type of approach for human vaccination has been set by malaria vaccine, which uses live attenuated sporozoites (Sanaria^®^ PfSPZ Vaccine) and has now completed phase 1/2a clinical trial (30). This study represents a meta-analysis of the experimental irradiated cercariae vaccine studies to identify robust variables that affect the levels of protection to inform human vaccine research and development.

The random effect meta-regression models identified three predictors of a reduction in worm burden: these were the total number of irradiated cercariae per immunization, the time between the last immunization and challenge, and the irradiation dose for parasite attenuation. We identified a positive correlation between the number of irradiated cercariae per immunization and the level of protection within the range of 50–5,000 cercariae used in the original studies. The models suggested that the optimally prepared irradiated cercariae vaccine could achieve a protection as high as 78% against challenge infection. As fitted graphs have shown, this is predicted for a single vaccination with 5,000 cercariae attenuated with 3 krad irradiation. This protection declines over time, but remains high for at least 8 months after the last immunization. Approximately 70% protection against challenge infection could be achieved after 8 months.

The number of immunizing cercariae represents the antigen dose, our results show a positive dose dependency of schistosome attenuated vaccine for higher protection. Studies of live attenuated vaccine for malaria infection also reported a similar positive correlation between the dose of immunizing parasites and the level of protection against future infection. Recently, as part of the phase 1 clinical trial of the human malaria vaccine using live attenuated sporozoites (Sanaria^®^ PfSPZ Vaccine), a dose-escalation trial was conducted using 7,500–135,000 irradiated *Plasmodium falciparum* sporozoites per immunization. The participants group that received the highest dose per immunization achieved the highest levels of protection against challenge infection (31–33). Although the adequate number of immunizing schistosome parasites are needed to protect baboon hosts has not been well quantified yet, experimental studies have been conducted with up to 45,000 schistosome parasites and reported positive protections (34–36). These reports suggest that a large number of attenuated cercariae would be required for vaccination in humans. The intermediate host snails have been reported to shed a large number of cercariae that is approximately 3,600–6,000 cercariae per snail over the first 50 days of shedding (37). Schistosome infected snails and cercariae are commercially available from organizations such as the NIH-NIAID Schistosomiasis Resource Center (38) and Schistosomiasis Collection at the Museum at National History Museum, London for laboratory use (39). Clearly producing an adequate number of cercariae of a satisfactory quality to use in vaccinations is still highly challenging (18). Although we cannot directly translate animal vaccine study results into human use, their value is in highlighting the nature and development of vaccine induced protective immunity against schistosomiasis. For example, the dynamic relationships between vaccination dose and level of protection are informative for human studies, as has been alluded to by drug induced resistance against re-infection (40, 41). It is also worth mentioning that human vaccination trials using infection or irradiated parasite vaccination have recently been conducted in human *P. falciparum* studies (42–44).

The result from the random effects meta-regression model showed a decrease in the fraction of protection with an increased time between the last immunization and challenge. This period between immunization and challenge represents the time to secondary encounter with the same antigen. When the initial encounter with the antigen takes place via infection or vaccination, the number of B and T cell produced against the antigen increases dramatically (45–48). Only a small fraction of those cells will survive as antigen-specific memory T and B cells or as long-lived plasma cells and they will be maintained for a long time (45–48). The duration of immune memory in humans after the vaccination is still controversial (49). However, there are several reports estimates for the length of immune memory after the last booster vaccination; 15 years for combined hepatitis A and B vaccine (50), 22 years for hepatitis B vaccine (51), over 30 years for poliovirus vaccine (49, 52), and over 60 years for small pox vaccine (49, 53). A longitudinal immuno-epidemiological study of schistosomiasis has been conducted by Butterworth et al., which reported that the protection induced by chemotherapy can remain for as long as 21 months after the treatment (54). However, other studies reported treated participants’ re-infection within 1 year (12, 55). One of the difficulties in evaluating the length of protective immunity in humans is that, in contrast to experimental animals, humans encounter a variety of antigens that could stimulate their immune systems through their daily life. In addition, people infected and being treated for schistosomiasis normally live in schistosomiasis endemic areas. Regarding the influence of schistosome infection on vaccine efficacy, Kariuki et al. have shown that the protection levels induced by attenuated cercariae vaccination were high in baboon hosts in a group chronically infected and then treated after vaccination, as well as in a group that was infected and previously treated before vaccination (36). In addition, encounters with infectious cercariae by people in endemic areas may work as a “natural booster” to stimulate protective immunity. In our study, the times between the last immunization and challenge (7–230 days) were relatively short compared with the life span of humans and schistosome parasites. This reflects that the average lifespan of a mouse is much shorter than that of the schistosome parasite (56, 57). The decrease in the fraction of protection over time was captured with our models even within this relatively short time range. This result would suggest that boosting vaccines may be necessary for long lasting protection against schistosomes.

There are several different cercariae attenuation methods as we described in the introduction. Within these, ionizing radiation (X-ray and gamma ray) is the most commonly used attenuation method for attenuated schistosome cercariae preparation. Two relatively high irradiation doses around 20 or 50 krad have been reported as the optimal doses for parasite attenuation (58, 59) and, in fact, many past studies have applied these irradiation doses. However, our results suggest that a lower irradiation dose could improve protection. The lower irradiation doses enable attenuated parasites to live longer in the vaccinated host. After vaccination, irradiated cercariae have been reported to be present around the skin exposure site for approximately 4 days and then gradually moved to the lungs where they transformed from cercariae into lung-stage schistosomula (60). It has been reported that the immunizing parasite has to reach the lungs and transform to lung-stage schistosomula to elicit protective immunity against challenge infection (60, 61), which may not be the case for cercariae attenuated with high doses of ionizing radiation. Several studies have reported that non-attenuated challenge cercariae in vaccinated mice slowly move to the lungs and then gradually disappear (61, 62). Several studies report that cercariae exposed to extremely high irradiation doses will die in the host skin before they start to migrate inside the host body (60, 63). Mountford et al. reported that hosts needed to be exposed to both highly irradiated cercariae, which die in the host skin, and lung-stage schistosome parasites to elicit protective immunity (64). One of the possible reasons for the high levels of protection observed when using irradiated cercariae vaccine is that hosts are exposed to a wide variety of antigens, which are expressed by different parasite life stages. Parasites, which were attenuated with lower irradiation dose, can survive long enough to express a variety of antigens from different life stages (65). However, in practice, allowing parasites to live longer inside vaccinated people may not be well accepted or ethically approved. The results of our meta-analysis suggests that for recombinant vaccine development the combination of antigens, which are unique to the different schistosome life stages may be an important factor in achieving a better protection.

## Conclusion

In this study, we identified three predictors for effective immunization against schistosome infection using attenuated cercariae: the total number of immunizing parasites, the irradiation dose, and the time between the last immunization and challenge. The study results suggested that the optimally prepared irradiated cercariae vaccine could achieve a protection as high as 78% against challenge infection. Within the reported dose range, the maximum protection could be achieved with the highest number of immunizing cercariae (5,000 cercariae) and the lowest irradiation dose (3 krad). This protection slowly declines but remains high for at least 8 months after the last immunization. This achievable protection is much higher than the partial protection reported by the *S. mansoni* purified antigens that failed to achieve consistent protection above 40% in mice (21, 66, 67). Although none of the radiation attenuated cercariae vaccine studies achieved complete protection against challenge infection, it is thought that partial protection induced by immunization can significantly reduce both schistosome related morbidity and parasite transmission (68, 69). This meta-analysis shows there is the high potential for an attenuated cercarial vaccine, while also providing insights helpful for schistosome vaccine development more generally.

## Author Contributions

The initial conception and design of the work: KM, CB, and FM. Performed the systematic review: MF, KM, and CB. Contributed to draft manuscript editing/reviewing: MF, KM, CB, MW, and FM. Statistical analyses of the data: MF, with inputs from MW, and FM. All authors contributed to the revisions and approved the final version of the manuscript.

## Conflict of Interest Statement

The authors declare that the research was conducted in the absence of any commercial or financial relationships that could be construed as a potential conflict of interest.

## Supplementary Material

The Supplementary Material for this article can be found online at http://www.frontiersin.org/Journal/10.3389/fimmu.2015.00085/abstract

## Footnotes

^1^http://www.webofknowledge.com

^2^http://www.elsevier.com

^3^http://www.ovid.com

^4^http://ovidsp.tx.ovid.com

^5^http://www.proquest.com
