# Supplementary material for: A Meta-Analysis of Experimental Studies of Attenuated Schistosoma mansoni Vaccines in the Mouse Model
Source: Front Immunol. 2015 Feb 27;6:85. doi: 10.3389/fimmu.2015.00085 (PMC4343029; doi:10.3389/fimmu.2015.00085)
Supplement: Supplementary file 1 [file Table_1.PDF]

Appendix
List of articles included for the analysis (1-105)

1. Agnew AM, Murare HM, Doenhoff MJ. Specific cross-protection between *Schistosoma bovis* and *S. haematobium* induced by highly irradiated infections in mice. *Parasite Immunol* (1989) **11**(4):341-9. doi: DOI 10.1111/j.1365-3024.1989.tb00672.x.
2. Aitken R, Coulson PS, Dixon B, Wilson RA. Radiation-resistant acquired-immunity of vaccinated mice to *Schistosoma mansoni*. *Am J Trop Med Hyg* (1987) **37**(3):570-7.
3. Aitken R, Coulson PS, Wilson RA. Pulmonary leukocytic responses are linked to the acquired-immunity of mice vaccinated with irradiated cercariae of *Schistosoma mansoni*. *J Immunol* (1988) **140**(10):3573-9.
4. Anderson S, Coulson PS, Ljubojevic S, Mountford AP, Wilson RA. The radiation-attenuated schistosome vaccine induces high levels of protective immunity in the absence of B cells. *Immunology* (1999) **96**(1):22-8.
5. Barsoum IS, Bogitsh BJ, Colley DG. Detection of *Schistosoma mansoni* circulating cathodic antigen for evaluation of resistance induced by irradiated cercariae. *J Parasitol* (1992) **78**(4):681-6. doi: Doi 10.2307/3283545.
6. Beaudoin RL, Armstrong JC, Vannier WE. Production of radiation-attenuated vaccines against malaria and schistosomiasis. *Int J Nucl Med Biol* (1980) **7**(2):113-24. doi: Doi 10.1016/0047-0740(80)90029-7.
7. Bickle QD. Studies on the relationship between the survival of *Schistosoma mansoni* larvae in mice and the degree of resistance produced. *Parasitology* (1982) **84**(Feb):111-22.
8. Bickle QD, Andrews BJ. Resistance following drug attenuation (ro-11-3128 or oxamniquine) of early *Schistosoma mansoni* infections in mice. *Parasitology* (1985) **90**(Apr):325-38.
9. Bickle QD, Doenhoff MJ. Comparison of the live vaccine potential of different geographic isolates of *Schistosoma mansoni*. *J Helminthol* (1987) **61**(3):191-5.
10. Bickle QD, Ford MJ, Andrews BJ. Studies on the development of anti-schistosomular surface antibodies by mice exposed to irradiated cercariae, adults and/or eggs of *S. mansoni*. *Parasite Immunol* (1983) **5**(5):499-511. doi: DOI 10.1111/j.1365-3024.1983.tb00764.x.
11. Bickle QD, Sacko M, Vignali DAA. Induction of immunity against *Schistosoma mansoni* by drug (ro11-3128)-terminated infections: analysis of surface-antigen recognition. *Parasite Immunol* (1990) **12**(6):569-86. doi: DOI 10.1111/j.1365-3024.1990.tb00989.x.
12. Bickle QD, Taylor MG, Doenhoff MJ, Nelson GS. Immunization of mice with gamma-irradiated intramuscularly injected schistosomula of *Schistosoma mansoni*. *Parasitology* (1979) **79**(Oct):209-22.
13. Cheever AW, Hieny S, Duvall RH, Sher A. Lack of resistance to *Schistosoma japonicum* in mice immunized with irradiated *S. mansoni* cercariae. *Trans R Soc Trop Med Hyg* (1983) **77**(6):812-4.
14. Constant SL, Mountford AP, Wilson RA. Phenotypic analysis of the cellular responses in regional lymphoid organs of mice vaccinated against *Schistosoma mansoni*. *Parasitology* (1990) **101 Pt 1**:15-22.
15. Correa-Oliveira R, James SL, McCall D, Sher A. Identification of a genetic locus, Rsm-1, controlling protective immunity against *Schistosoma mansoni*. *J Immunol* (1986) **137**(6):2014-9.
16. Correa-Oliveira R, Sher A, James SL. Defective vaccine-induced immunity to *Schistosoma mansoni* in P strain mice. I. Analysis of antibody responses. *J Immunol* (1984) **133**(3):1581-6.
17. Coulson PS, Mountford AP. Fate of attenuated schistosomula administered to mice by different routes, relative to the immunity induced against *Schistosoma mansoni*. *Parasitology* (1989) **99 Pt 1**:39-45.
18. Coulson PS, Wilson RA. Examination of the mechanisms of pulmonary phase resistance to *Schistosoma mansoni* in vaccinated mice. *Am J Trop Med Hyg* (1988) **38**(3):529-39.
19. Coulson PS, Wilson RA. Recruitment of lymphocytes to the lung through vaccination enhances the immunity of mice exposed to irradiated schistosomes. *Infect Immun* (1997) **65**(1):42-8.
20. Crabtree JE, Wilson RA. The role of pulmonary cellular reactions in the resistance of vaccinated mice to *Schistosoma mansoni*. *Parasite Immunol* (1986) **8**(3):265-85.

21. Dean DA, Bukowski MA, Clark SS. Attempts to transfer the resistance of *Schistosoma mansoni* infected and irradiated cercaria-immunized mice by means of parabiosis. *Am J Trop Med Hyg* (1981) **30**(1):113-20.
22. Dean DA, Cioli D, Bukowski MA. Resistance induced by normal and irradiated *Schistosoma mansoni*: ability of various worm stages to serve as inducers and targets in mice. *Am J Trop Med Hyg* (1981) **30**(5):1026-32.
23. Dean DA, Mangold BL. Evidence that both normal and immune elimination of *Schistosoma mansoni* take place at the lung stage of migration prior to parasite death. *Am J Trop Med Hyg* (1992) **47**(2):238-48.
24. Dean DA, Mangold BL, Georgi JR, Jacobson RH. Comparison of *Schistosoma mansoni* migration patterns in normal and irradiated cercaria-immunized mice by means of autoradiographic analysis: evidence that worm elimination occurs after the skin phase in immunized mice. *Am J Trop Med Hyg* (1984) **33**(1):89-96.
25. Dean DA, Mangold BL, Lewis FA. Comparison of two strains of *Schistosoma mansoni* with respect to the sites and kinetics of immune elimination in irradiated cercaria-immunized mice. *J Parasitol* (1995) **81**(1):43-7.
26. Dean DA, Murrell KD, Xu ST, Mangold BL. Immunization of mice with ultraviolet-irradiated *Schistosoma mansoni* cercariae: a re-evaluation. *Am J Trop Med Hyg* (1983) **32**(4):790-3.
27. Delgado VS, Mclaren DJ. Evidence that radio-sensitive cells are central to skin-phase protective immunity in CBA/Ca mice vaccinated with radiation-attenuated cercariae of *Schistosoma mansoni* as well as in naive mice protected with vaccine serum. *Parasitology* (1990) **100**:45-56.
28. Dent LA, Munro GH, Piper KP, Sanderson CJ, Finlay DA, Dempster RK, et al. Eosinophilic interleukin 5 (IL-5) transgenic mice: Eosinophil activity and impaired clearance of *Schistosoma mansoni*. *Parasite Immunol* (1997) **19**(7):291-300. doi: DOI 10.1046/j.1365-3024.1997.d01-210.x.
29. El Ridi R, Tawab NA, Guirguis N. *Schistosoma mansoni*: identification and protective immunity of adult worm antigens recognized by T lymphocytes of outbred swiss mice immunized with irradiated cercariae. *Exp Parasitol* (1993) **76**(3):265-77. doi: DOI 10.1006/expr.1993.1032.
30. Erickson DG, Caldwell WL. Acquired resistance in mice and rats after exposure to gamma-irradiated cercariae. *Am J Trop Med Hyg* (1965) **14**(4):566-73.
31. Flisser A, Delgado VS, Mclaren DJ. *Schistosoma mansoni*: enhanced efficacy of praziquantel treatment in immune mice. *Parasite Immunol* (1989) **11**(4):319-28. doi: DOI 10.1111/j.1365-3024.1989.tb00670.x.
32. Hackett F, Simpson AJG, Omerali P, Smithers SR. Surface-antigens of and cross-protection between 2 geographical isolates of *Schistosoma mansoni*. *Parasitology* (1987) **94**:301-12.
33. Hanna LS, Mohamed MI, Botros SS. *Schistosoma mansoni*: IgG reactivity to soluble adult worm antigen preparation (SWAP) in sera of immunized mice with irradiated attenuated and virulent cercariae. *J Egypt Soc Parasitol* (1994) **24**(3):553-68.
34. Hewitson JP, Hamblin PA, Mountford AP. In the absence of CD154, administration of interleukin-12 restores Th1 responses but not protective immunity to *Schistosoma mansoni*. *Infect Immun* (2007) **75**(7):3539-47. doi: Doi 10.1128/Iai.00252-07.
35. Hsu SYL, Hsu HF, Burmeister LF. *Schistosoma mansoni*: vaccination of mice with highly x-irradiated cercariae. *Exp Parasitol* (1981) **52**(1):91-104.
36. Hsu SYL, Hsu HF, Osborne JW, Johnson SC. *Schistosoma mansoni*: is acquired-immunity induced by highly X-irradiated cercariae dependent on the size of the challenging dose. *J Parasitol* (1982) **68**(2):199-201. doi: Doi 10.2307/3281174.
37. James ER, Dobinson AR. Comparison of the protective resistance induced by 60Co-irradiated cercariae and schistosomula of the WFFS and NMRI strains of *Schistosoma mansoni*. *J Helminthol* (1985) **59**(4):313-7.
38. James SL, Sher A. Mechanisms of protective immunity against *Schistosoma mansoni* infection in mice vaccinated with irradiated cercariae .3. Identification of a mouse strain, P/N, that fails to respond to vaccination. *Parasite Immunol* (1983) **5**(6):567-75. doi: DOI 10.1111/j.1365-3024.1983.tb00773.x.

39. Kambara T, Wilson RA. In situ pulmonary responses of T cell and macrophage subpopulations to a challenge infection in mice vaccinated with irradiated cercariae of *Schistosoma mansoni*. *J Parasitol* (1990) **76**(3):365-72.
40. Kamiya H, Smithers SR, McLaren DJ. *Schistosoma mansoni*: autoradiographic tracking studies of isotopically-labelled challenge parasites in naive and vaccinated CBA/Ca mice. *Parasite Immunol* (1987) **9**(4):515-29.
41. Kelly EA, Colley DG. Effects of immunomanipulations on resistance induced by irradiated *Schistosoma mansoni* cercarial sensitization of C57BL/6 and CBA/J mice. *Am J Trop Med Hyg* (1986) **35**(4):803-11.
42. Kelly EA, Colley DG. In vivo effects of monoclonal anti-L3T4 antibody on immune responsiveness of mice infected with *Schistosoma mansoni*. Reduction of irradiated cercariae-induced resistance. *J Immunol* (1988) **140**(8):2737-45.
43. King CL, Jia XL, Malhotra I, Liu SF, Mahmoud AAF, Oettgen HC. Mice with a targeted deletion of the IgE gene have increased worm burdens and reduced granulomatous inflammation following primary infection with *Schistosoma mansoni*. *J Immunol* (1997) **158**(1):294-300.
44. King CL, Malhotra I, Jia XL. *Schistosoma mansoni*: Protective immunity in IL-4-deficient mice. *Exp Parasitol* (1996) **84**(2):245-52. doi: 10.1006/expr.1996.0110.
45. Lewis FA, Wilson EM. *Schistosoma mansoni*: splenic lymphocyte-responses of mice after initial exposure to highly irradiated cercariae. *Exp Parasitol* (1982) **54**(1):21-32. doi: 10.1016/0014-4894(82)90106-0.
46. Lewis FA, Winestock J, Dingaan B, Richards C, Dean DA. Intraspecific cross-protection in mice immunized with irradiated *Schistosoma mansoni* cercariae. *J Parasitol* (1987) **73**(4):787-91. doi: 10.2307/3282414.
47. Mangold BL, Dean DA. The migration and survival of gamma-irradiated *Schistosoma mansoni* larvae and the duration of host-parasite contact in relation to the induction of resistance in mice. *Parasitology* (1984) **88**(Apr):249-66.
48. Mangold BL, Dean DA, Coulson PS, Wilson RA. Site requirements and kinetics of immune-dependent elimination of intravascularly administered lung stage schistosomula in mice immunized with highly irradiated cercariae of *Schistosoma mansoni*. *Am J Trop Med Hyg* (1986) **35**(2):332-44.
49. Mastin AJ, Bickle QD, Wilson RA. *Schistosoma mansoni*: migration and attrition of irradiated and challenge schistosomula in the mouse. *Parasitology* (1983) **87**(Aug):87-102.
50. Mclaren DJ, Pearce EJ, Smithers SR. Site potential for challenge attrition in mice, rats and guinea-pigs vaccinated with irradiated cercariae of *Schistosoma mansoni*. *Parasite Immunol* (1985) **7**(1):29-44. doi: 10.1111/j.1365-3024.1985.tb00477.x.
51. Mclaren DJ, Smithers SR. Serum from CBA/Ca mice vaccinated with irradiated cercariae of *Schistosoma mansoni* protects naive recipients through the recruitment of cutaneous effector-cells. *Parasitology* (1988) **97**:287-302.
52. Mclaren DJ, Strath M, Smithers SR. *Schistosoma mansoni*: evidence that immunity in vaccinated and chronically infected CBA/Ca mice is sensitive to treatment with a monoclonal-antibody that depletes cutaneous effector-cells. *Parasite Immunol* (1987) **9**(6):667-82. doi: 10.1111/j.1365-3024.1987.tb00537.x.
53. Menson EN, Coulson PS, Wilson RA. *Schistosoma mansoni*: circulating and pulmonary leucocyte responses related to the induction of protective immunity in mice by irradiated parasites. *Parasitology* (1989) **98 (Pt 1)**:43-55.
54. Menson EN, Wilson RA. Lung-phase immunity to *Schistosoma mansoni*. Flow cytometric analysis of macrophage activation states in vaccinated mice. *J Immunol* (1989) **143**(7):2342-8.
55. Menson EN, Wilson RA. Lung-phase immunity to *Schistosoma mansoni*: definition of alveolar macrophage phenotypes after vaccination and challenge of mice. *Parasite Immunol* (1990) **12**(4):353-66.
56. Miller KL, Smithers SR. *Schistosoma mansoni*: the attrition of a challenge infection in mice immunized with highly irradiated live cercariae. *Exp Parasitol* (1980) **50**(2):212-21. doi: 10.1016/0014-4894(80)90022-3.

57. Miller KL, Smithers SR. Localized skin changes at the site of immunization with highly irradiated cercariae of *Schistosoma mansoni* are associated with enhanced resistance to a challenge infection. *Parasitology* (1982) **85**(Oct):305-14.
58. Miller KL, Smithers SR, Sher A. The response of mice immune to *Schistosoma mansoni* to a challenge infection which bypasses the skin: evidence for 2 mechanisms of immunity. *Parasite Immunol* (1981) **3**(1):25-31. doi: 10.1111/j.1365-3024.1981.tb00382.x.
59. Minard P, Dean DA, Jacobson RH, Vannier WE, Murrell KD. Immunization of mice with cobalt-60 irradiated *Schistosoma mansoni* cercariae. *Am J Trop Med Hyg* (1978) **27**(1 Pt 1):76-86.
60. Minard P, Dean DA, Vannier WE, Murrell KD. Effect of immunization on migration of *Schistosoma mansoni* through lungs. *Am J Trop Med Hyg* (1978) **27**(1):87-93.
61. Mitchell GF, Davern KM, Wood SM, Wright MD, Argyropoulos VP, Mcleod KS, et al. Attempts to induce resistance in mice to *Schistosoma japonicum* and *Schistosoma mansoni* by exposure to crude schistosome antigens plus cloned glutathione-S-transferases. *Immunol Cell Biol* (1990) **68**:377-85. doi: 10.1038/Icb.1990.51.
62. Moloney NA, Bickle QD, Webbe G. The induction of specific immunity against *Schistosoma japonicum* by exposure of mice to ultraviolet attenuated cercariae. *Parasitology* (1985) **90**(Apr):313-23.
63. Mountford AP, Coulson PS, Pemberton RM, Smythies LE, Wilson RA. The generation of interferon-gamma-producing lymphocytes-t in skin-draining lymph-nodes, and their recruitment to the lungs, is associated with protective immunity to *Schistosoma mansoni*. *Immunology* (1992) **75**(2):250-6.
64. Mountford AP, Coulson PS, Wilson RA. Antigen localization and the induction of resistance in mice vaccinated with irradiated cercariae of *Schistosoma mansoni*. *Parasitology* (1988) **97**:11-25.
65. Mountford AP, Hogg KG, Coulson PS, Brombacher F. Signaling via interleukin-4, receptor alpha chain is required for successful vaccination against schistosomiasis in BALB/c mice. *Infect Immun* (2001) **69**(1):228-36. doi: 10.1128/Iai.69.1.228-236.2001.
66. Mountford AP, Wilson RA. *Schistosoma mansoni*: the effect of regional lymphadenectomy on the level of protection induced in mice by radiation-attenuated cercariae. *Exp Parasitol* (1990) **71**(4):463-9. doi: 10.1016/0014-4894(90)90072-K.
67. Murrell KD, Clark S, Dean DA, Vannier WE. Influence of mouse strain on induction of resistance with irradiated *Schistosoma mansoni* cercariae. *J Parasitol* (1979) **65**(5):829-31. doi: 10.2307/3280378.
68. Navarrete S, Rollinson D, Agnew AM. Cross-protection between species of the *Schistosoma haematobium* group induced by vaccination with irradiated parasites. *Parasite Immunol* (1994) **16**(1):19-25. doi: 10.1111/j.1365-3024.1994.tb00300.x.
69. Noonan FP, Lewis FA. UVB-induced immune suppression and infection with *Schistosoma mansoni*. *Photochem Photobiol* (1995) **61**(1):99-105.
70. Oligino LD, Percy AJ, Harn DA. Purification and immunochemical characterization of a 22 kilodalton surface antigen from *Schistosoma mansoni*. *Mol Biochem Parasitol* (1988) **28**(2):95-103. Epub 1988/03/01. doi: 10.1016/0166-6851(88)90056-4
71. Pemberton RM, Wilson RA. T-helper type-1-dominated lymph node responses induced in C57BL/6 mice by optimally irradiated cercariae of *Schistosoma mansoni* are down-regulated after challenge infection. *Immunology* (1995) **84**(2):310-6.
72. Piper KP, McLaren DJ. The role of T cells in vaccine immunity in the murine model of *Schistosomiasis mansoni*. *Int J Parasitol* (1993) **23**(2):245-56.
73. Pons HA, Morgan JS, Hutchinson ML, Rojkind M, Groszmann RJ, Stadecker MJ. Resistance to reinfection in experimental murine schistosomiasis: role of porto-hepatic hemodynamics. *Am J Trop Med Hyg* (1989) **41**(2):189-97.
74. Radke MG, Sadun EH. Resistance produced in mice by exposure to irradiated *Schistosoma mansoni* cercariae. *Exp Parasitol* (1963) **13**:134-42.
75. Ratcliffe EC, Wilson RA. The magnitude and kinetics of delayed-type hypersensitivity responses in mice vaccinated with irradiated cercariae of *Schistosoma mansoni*. *Parasitology* (1991) **103 Pt 1**:65-75.

76. Ratcliffe EC, Wilson RA. The role of mononuclear-cell recruitment to the lungs in the development and expression of immunity to *Schistosoma mansoni*. *Parasitology* (1992) **104**:299-307.
77. Reynolds SR, Harn DA. Comparison of irradiated-cercaria schistosome vaccine models that use 15 kilorad and 50 kilorad doses: the 15 kilorad dose gives greater protection, smaller liver sizes, and higher gamma-interferon levels after challenge. *Infect Immun* (1992) **60**(1):90-4.
78. Richter D, Incani RN, Harn DA. Isotype responses to candidate vaccine antigens in protective sera obtained from mice vaccinated with irradiated cercariae of *Schistosoma mansoni*. *Infect Immun* (1993) **61**(7):3003-11.
79. Ruppel A, Rother U, Vongerichten H, Lucius R, Diesfeld HJ. *Schistosoma mansoni*: immunoblot analysis of adult worm proteins. *Exp Parasitol* (1985) **60**(2):195-206. Epub 1985/10/01. doi: 10.1016/0014-4894(85)90023-2
80. Sadun EH. Immunization in schistosomiasis by previous exposure to homologous and heterologous cercariea by inoculation of preparations from schistosomes and by exposure to irradiated cercariae. *Ann N Y Acad Sci* (1963) **113**:418-39.
81. Sher A, Benno D. Decreasing immunogenicity of developing schistosome larvae. *Parasite Immunol* (1982) **4**(2):101-7.
82. Sher A, Coffman RL, Hieny S, Cheever AW. Ablation of eosinophil and IgE responses with anti-IL-5 or anti-IL-4 antibodies fails to affect immunity against *Schistosoma mansoni* in the mouse. *J Immunol* (1990) **145**(11):3911-6.
83. Sher A, Correa-Oliveira R, Hieny S, Hussain R. Mechanisms of protective immunity against *Schistosoma mansoni* infection in mice vaccinated with irradiated cercariae. IV. Analysis of the role of IgE antibodies and mast cells. *J Immunol* (1983) **131**(3):1460-5.
84. Sher A, Hieny S, James S. Mechanisms of protective immunity against *S. mansoni* infection in mice vaccinated with irradiated cercariae. VI. Influence of the major histocompatibility complex. *Parasite Immunol* (1984) **6**(4):319-28.
85. Sher A, Hieny S, James SL, Asofsky R. Mechanisms of protective immunity against *Schistosoma mansoni* infection in mice vaccinated with irradiated cercariae. II. Analysis of immunity in hosts deficient in T lymphocytes, B lymphocytes, or complement. *J Immunol* (1982) **128**(4):1880-4.
86. Simpson AJ, Hackett F, Walker T, de Rossi R, Smithers SR. Antibody response against schistosomulum surface antigens and protective immunity following immunization with highly irradiated cercariae of *Schistosoma mansoni*. *Parasite Immunol* (1985) **7**(2):133-52.
87. Smith MA, Clegg JA. *Schistosoma mansoni*: decay of resistance induced by gamma irradiated cercariae in the mouse. *Trans R Soc Trop Med Hyg* (1984) **78**(2):190-2.
88. Smythies LE, Coulson PS, Wilson RA. Monoclonal antibody to IFN-gamma modifies pulmonary inflammatory responses and abrogates immunity to *Schistosoma mansoni* in mice vaccinated with attenuated cercariae. *J Immunol* (1992) **149**(11):3654-8.
89. Smythies LE, Pemberton RM, Coulson PS, Mountford AP, Wilson RA. T cell-derived cytokines associated with pulmonary immune mechanisms in mice vaccinated with irradiated cercariae of *Schistosoma mansoni*. *J Immunol* (1992) **148**(5):1512-8.
90. Stek M, Jr., Dean DA, Clark SS. Attrition of schistosomes in an irradiation-attenuated cercarial immunization model of *Schistosoma mansoni*. *Am J Trop Med Hyg* (1981) **30**(5):1033-8.
91. Stek M, Jr., Minard P, Cruess DF. Murine immunization by cesium-137 irradiation attenuated *Schistosoma mansoni* cercariae. *J Parasitol* (1984) **70**(3):398-402.
92. Stek M, Sulaiman SM. Thermal effects on *Schistosoma mansoni* irradiation-attenuated vaccine production and administration. *P Helm Soc Wash* (1984) **51**(2):287-92.
93. Stek MF, Minard P, Dean DA, Hall JE. Immunization of baboons with *Schistosoma mansoni* cercariae attenuated by gamma irradiation. *Science* (1981) **212**(4502):1518-20.
94. Street M, Coulson PS, Sadler C, Warnock LJ, McLaughlin D, Bluethmann H, et al. TNF is essential for the cell-mediated protective immunity induced by the radiation-attenuated schistosome vaccine. *J Immunol* (1999) **163**(8):4489-94.
95. Szumlewicz AP, Olivier LJ. *Schistosoma mansoni*: development of challenge infections in mice exposed to irradiated cercariae. *Science* (1963) **140**(3565):411-2.

96. Tawfik AF, Colley DG. Effects of anti-schistosomal chemotherapy on immune responses, protection and immunity. II. Concomitant immunity and immunization with irradiated cercariae. *Am J Trop Med Hyg* (1986) **35**(1):110-7.
97. Timothy LM, Coulson PS, Behnke JM, Wilson RA. Cross-reactivity between *Necator americanus* and *Schistosoma mansoni* in mice. *Int J Parasitol* (1992) **22**(8):1143-9. doi: 10.1016/0020-7519(92)90033-H.
98. Vignali DA, Bickle QD, Taylor MG, Tennent G, Pepys MB. Comparison of the role of complement in immunity to *Schistosoma mansoni* in rats and mice. *Immunology* (1988) **63**(1):55-61.
99. Vignali DA, Crocker P, Bickle QD, Cobbold S, Waldmann H, Taylor MG. A role for CD4+ but not CD8+ T cells in immunity to *Schistosoma mansoni* induced by 20 krad-irradiated and Ro 11-3128-terminated infections. *Immunology* (1989) **67**(4):466-72.
100. Ward REM, Mclaren DJ. *Schistosoma mansoni*: evidence that eosinophils and or macrophages contribute to skin-phase challenge attrition in vaccinated CBA/Ca mice. *Parasitology* (1988) **96**:63-84.
101. Wilson RA, Coulson PS, Betts C, Dowling MA, Smythies LE. Impaired immunity and altered pulmonary responses in mice with a disrupted interferon-gamma receptor gene exposed to the irradiated *Schistosoma mansoni* vaccine. *Immunology* (1996) **87**(2):275-82.
102. Wilson RA, Coulson PS, Dixon B. Migration of the schistosomula of *Schistosoma mansoni* in mice vaccinated with radiation-attenuated cercariae, and normal mice: an attempt to identify the timing and site of parasite death. *Parasitology* (1986) **92 (Pt 1)**:101-16.
103. Wynn TA, Jankovic D, Hieny S, Cheever AW, Sher A. IL-12 enhances vaccine-induced immunity to *Schistosoma mansoni* in mice and decreases T helper 2 cytokine expression, IgE production, and tissue eosinophilia. *J Immunol* (1995) **154**(9):4701-9.
104. Wynn TA, Oswald IP, Eltoum IA, Caspar P, Lowenstein CJ, Lewis FA, et al. Elevated expression of Th1 cytokines and nitric oxide synthase in the lungs of vaccinated mice after challenge infection with *Schistosoma mansoni*. *J Immunol* (1994) **153**(11):5200-9.
105. Wynn TA, Reynolds A, James S, Cheever AW, Caspar P, Hieny S, et al. IL-12 enhances vaccine-induced immunity to schistosomes by augmenting both humoral and cell-mediated immune responses against the parasite. *J Immunol* (1996) **157**(9):4068-78.
